# Graphene oxide improves postoperative cognitive dysfunction by maximally alleviating amyloid beta burden in mice

**DOI:** 10.7150/thno.50616

**Published:** 2020-10-25

**Authors:** Jiqian Zhang, Shasha Zhu, Peipei Jin, Yuting Huang, Qingqing Dai, Qianyun Zhu, Pengfei Wei, Zhilai Yang, Lei Zhang, Hu Liu, Guanghong Xu, Lijian Chen, Erwei Gu, Yunjiao Zhang, Longping Wen, Xuesheng Liu

**Affiliations:** 1 Department of Anesthesiology, the First Affiliated Hospital of Anhui Medical University, Key Laboratory of Anesthesiology and Perioperative Medicine of Anhui Higher Education Institutes, Anhui Medical University.; 2 Reproductive Medicine Center, Department of Obstetrics and Gynecology, the First Affiliated Hospital of Anhui Medical University; NHC Key Laboratory of Study on Abnormal Gametes and Reproductive Tract (Anhui Medical University); Key Laboratory of Population Health Across Life Cycle (Anhui Medical University), Ministry of Education of the People's Republic of China.; 3 The First Affiliated Hospital of USTC, Division of Life Sciences and Medicine, University of Science and Technology of China.; 4 School of Life Sciences, University of Science and Technology of China.; 5 Nanobio Laboratory, Institute of Life Sciences, South China University of Technology.

**Keywords:** graphene oxide, β-amyloid, postoperative cognitive dysfunction, fear memory

## Abstract

**Rationale:** Graphene oxide (GO) based nanomaterials have shown potential for the diagnosis and treatment of amyloid-β (Aβ)-related diseases, mainly on Alzheimer's disease (AD). However, these nanomaterials have limitations. How GO is beneficial to eliminate Aβ burden, and its physiological function in Aβ-related diseases, still needs to be investigated. Moreover, postoperative cognitive dysfunction (POCD) is an Aβ-related common central nervous system complication, however, nanomedicine treatment is lacking.

**Methods:** To evaluate the effects of GO on Aβ levels, HEK293T-APP-GFP and SHSY5Y-APP-GFP cells are established. Intramedullary fixation surgery for tibial fractures under inhalation anesthesia is used to induce dysfunction of fear memory in mice. The fear memory of mice is assessed by fear conditioning test.

**Results:** GO treatment maximally alleviated Aβ levels by simultaneously reducing Aβ generation and enhancing its degradation through inhibiting β-cleavage of amyloid precursor protein (APP) and improving endosomal Aβ delivery to lysosomes, respectively. In postoperative mice, the hippocampal Aβ levels were significantly increased and hippocampal-dependent fear memory was impaired. However, GO administration significantly reduced hippocampal Aβ levels and improved the cognitive function of the postoperative mice.

**Conclusion:** GO improves fear memory of postoperative mice by maximally alleviating Aβ accumulation, providing new evidence for the application of GO-based nanomedicines in Aβ-related diseases.

## Introduction

Postoperative cognitive dysfunction (POCD) is a common central nervous system complication after anesthesia and surgery, characterized by a decline in cognitive performance 1, 2. POCD is commonly observed in elderly patients, who often develop impaired concentration, memory, and learning after surgery 2. Currently, the decline in cognitive function is considered transient, and most patients can return to normal levels; however, a few patients can continue to suffer from it for a long time 3. POCD not only causes a significant economic problem and mental stress to patients and families, but also significantly increases the mortality rate of patients 4. The mechanism of POCD is complex, involving amyloid beta (Aβ) accumulation, hyperphosphorylation of tau protein, inflammation, synaptic functional deficit, and inhibition of central cholinergic transmission 5, 6. Consequently, Aβ burden is one of the most popular research subjects in POCD. Growing evidence suggests that anesthesia and surgery could enhance Aβ levels, and thus contribute to cognitive impairment and POCD occurrence 7-9. Therapeutic agents that attenuate brain Aβ accumulation are beneficial in rescuing cognitive impairment in aged mice after surgery 10. Additionally, in human cell lines, altered amyloid precursor protein (APP) processing and increased Aβ production were detected after isoflurane inhalation 11. These studies support the critical role of Aβ in the progression and pathology of POCD. Therefore, targeting Aβ could be an effective therapeutic method for POCD.

Aβ is a 39-43 amino acid peptide derived from the sequential cleavage of APP by α-, β-(BACE1), and γ-secretases 12. The cleavage and processing of APP can be divided into a non-amyloidogenic pathway and an amyloidogenic pathway. The amyloidogenic pathway, also known as an alternative cleavage pathway, leads to Aβ generation. In the amyloidogenic pathway, APP can be internalized into early endosomes, which contain BACE1 and have optimal pH for cleavage of APP. BACE1 cleavage of APP results in the generation of C-terminal fragment (β-CTF) within the membrane. Subsequently, γ-secretase cleaves β-CTF and liberates an intact Aβ peptide within the endosome/lysosome system. Finally, Aβ is transported to the lysosome and degraded 12. In addition, autophagy and the ubiquitin proteasome pathway (UPS) have also been reported to promote the degradation of Aβ 13, 14. Aβ can also be transported from the endosome to the cell surface or extracellular space. Moreover, Aβ that escapes degradation may be drained into the cerebrospinal fluid or cleared into the lymphatic or vascular circulation 15. Failure of these redundant turnover mechanisms will result in the accumulation and aggregation of Aβ, which initiates the pathogenic cascade that leads to tau protein hyperphosphorylation, intracellular neurofibrillary tangles, synaptic dysfunction, neuronal death, and ultimately, loss of cognitive function 16.

Nanomaterials, including inorganic nanoparticles, polymeric nanoparticles, carbon nanomaterials, and functionalization of nanomaterials, have shown great potential for the diagnosis and treatment of Aβ-related diseases 17, 18, mainly focusing on Alzheimer's disease (AD) 16, 19-23. The novel nanocarbon material, graphene oxide (GO)/graphene, has received much attention. Xiao *et al.* reported that graphene quantum dot conjugated neuroprotective peptide improved learning and memory in a mouse model of AD 24. Qu *et al.* showed that local and remote heat could dissociate amyloid aggregation via thioflavin-S (ThS)-modified GO with near infrared (NIR) laser irradiation 25. In addition, previous studies suggested that GO may be a promising inhibitor of Aβ assembly 26. GO has a strong interaction with Aβ33-42, thereby competitively reducing the inter-peptide interactions of Aβ33-42 peptides, further inhibiting its oligomerization and long mature fibril formation 26. These studies indicated that GO may be suitable for developing Aβ-targeted nanomedicines, however, the present study has some limitations. First, dissolving Aβ or merely inhibiting Aβ assembly by GO will result in a large amount of unaggregated-Aβ, which is still a great health risk. Secondly, the physiological mechanism of alleviating the Aβ burden by GO in Aβ-related diseases, such as POCD, remains unclear. Therefore, more evidence is needed to evaluate whether GO is suitable for the development of Aβ-targeted nanotherapy.

**Scheme 1:**
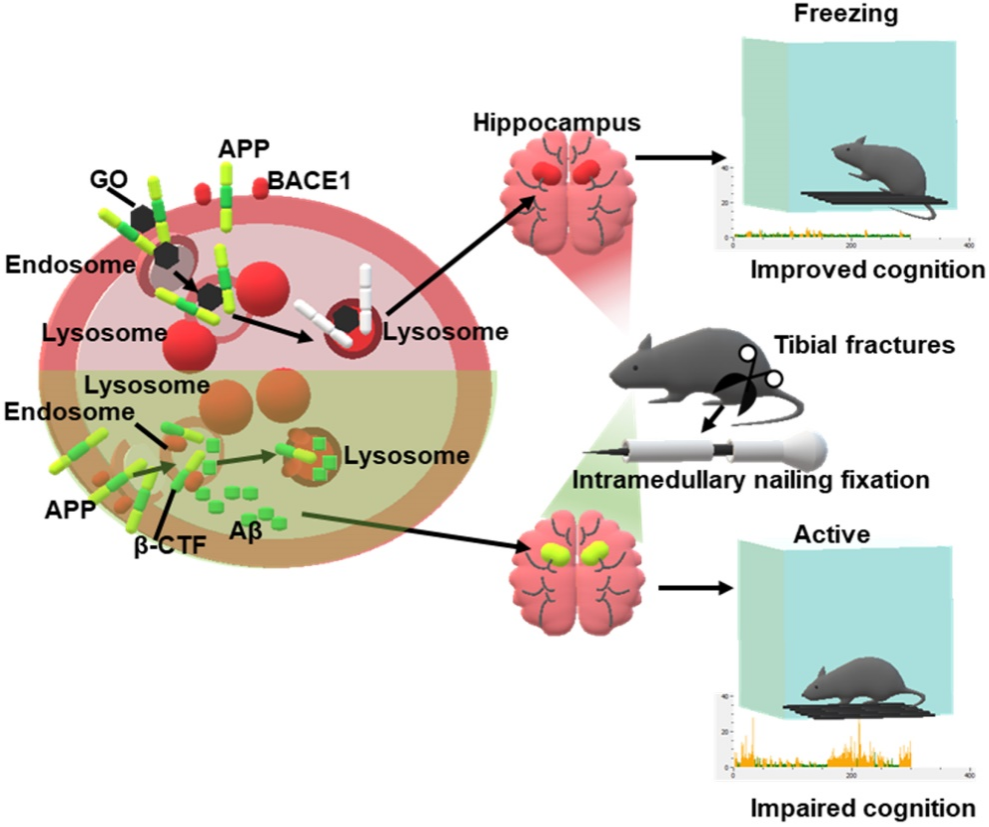
Schematic illustration of GO alleviating Aβ burden and improving postoperative cognitive dysfunction in mice. GO and APP had a higher affinity than APP and BACE1, resulting in less BACE1 on the endosomes, which further inhibited β-cleavage of APP and reduced the generation of Aβ. Moreover, GO improved endosomal APP/β-CTF and Aβ delivery to lysosomes and enhanced their degradation. The above effects synergistically contributed to the decrease of Aβ levels. Intramedullary fixation surgery for tibial fracture and anesthesia acutely increased the hippocampal β-CTF and Aβ levels and impaired hippocampal-dependent fear memory, however, administration of GO alleviated Aβ levels and improved the cognitive function of the mice.

Prior to intervening in Aβ assembly, reducing Aβ production and/or increasing Aβ degradation can maximally alleviate Aβ accumulation at its source. As such, we tested the effects of GO on the production and degradation of Aβ. Our results showed that GO treatment resulted in the decrease of Aβ levels in HEK293T-APP and SHSY5Y-APP cells. GO interacted with APP and disrupted the co-localization of APP and BACE1 on the endosomes, which further inhibited β-cleavage of APP and reduced the generation of Aβ. Moreover, GO increased the number of cellular endosomes, improved endosomal Aβ delivery to lysosomes, and enhanced its degradation. These effects synergistically contributed to the maximal alleviation of Aβ burden by GO. Furthermore, we constructed a mouse model of fear memory dysfunction by performing intramedullary fixation surgery for tibial fractures under inhalation anesthesia, which is commonly used in rodents 27-29. We found that surgery and anesthesia acutely increased hippocampal β-CTF and Aβ levels and impaired hippocampal-dependent fear memory in 10-month-old mice. However, GO administration significantly decreased Aβ levels and improved the cognitive function of mice. These results revealed that GO could improve impaired fear memory by maximally alleviating Aβ burden in mice (Scheme 1), providing new evidence for the application of GO-based nanomedicines in Aβ-related diseases.

## Materials and Methods

### Preparation and Characterization of GO

GO was prepared from purified graphite (purchased from Sigma-Aldrich) by the modified Hummers method 30, 31. First, graphite powder (1 g) and NaNO_3_ (1 g) were added to cooled concentrated H_2_SO_4_ (23 mL), and the mixture was stirred for 30 min in an ice bath. Then KMnO_4_ (3 g) was added gradually with stirring and cooling, A homogeneous liquid suspension was obtained after sonication for 5 h at 40 kHz to exfoliate the graphene oxide, after which deionized water was added gradually (46 mL over 15 min) and further stirred for 10 min. The reaction was terminated by adding deionized water (140 mL) and aqueous H_2_O_2_ (30%, 10 mL). The solid product was separated by centrifugation (1500 rpm, 10 min). Finally, the sediment was washed 5 times by suspending in HCl solution (5%), followed by washing twice with deionized water and then dehydrated. The resultant GO powder was prepared via tip sonication (Misonix Sonicator 3000) in an ice bath at a power of 380 W for 2 h.

The surface morphology of GO was characterized with an atomic force microscope (AFM, XE‐70, Park System) in tapping mode using the aluminum coating silicon probe (frequency 300 kHz, spring constants 40 N/m, scanning rate 1 Hz), under ambient conditions and scanning line of 512. The Fourier transform infrared (ATR‐FTIR) spectra of GO were recorded using a Perkine Elmer Spectrum RXI FTIR spectrometer with 2 cm^‐1^ resolution and 32 scans, and the background was collected in the absence of samples. The size distribution of GO was characterized by using Dynamic Light Scattering (380 ZLS, Nicomp, USA) from Particle Sizing Systems at room temperature. UV‐vis spectra were measured with a Hitachi U‐2810 UV‐visible spectrophotometer equipped with a 10 mm quartz cell, and 1 cm light path length at room temperature. The surface concentrations of C and O were measured by X‐ray photoelectron spectroscopy (XPS, ESCALAB 250).

### Cell culture and establishment of APP overexpressed cell line

HKE293T and SHSY5Y Cells were cultured at 37 °C with 5% CO_2_ in Dulbecco's Modified Eagle's Medium supplemented with 10% fetal bovine serum (FBS). pLenti-CMV-APP-GFP-Puro (purchased from Public Protein Library) or empty vector with pMD2.G and pPsAX2.0 were transfected into HEK293T293T cells with Lipofectamine 3000 for production of lentiviruses. After 2 d, SHSY5Y cells were infected with the filtered lentiviruses in medium containing 5 μg/mL polybrene. The transduced cells were selected with 1 μg/mL puromycin for 4 d.

### Immunofluorescence

Cells were fixed using 4% paraformaldehyde for 10 min, permeabilized with 0.1% Triton X-100 for 10 min, and blocked with 1% FBS for 1 h. Cells were incubated with primary antibodies overnight at 4 °C and labeled with secondary antibodies at 37 °C for 1 h. Images were acquired using confocal fluorescence microscopy (LSM800, ZEISS). For making frozen sections, mice were anesthetized with sodium pentobarbital and perfused with PBS, followed by 4% paraformaldehyde. Following perfusion, the brain was obtained and post-fixed overnight in fixative solution, then cryoprotected overnight in 30% sucrose in PBS. Frozen brain tissues were embedded in TissueTek OCT compound, then cut into 10 μm sections. The sections were observed under confocal fluorescence microscopy (LSM800, ZEISS). BACE1 (1: 200, 3C1C3; 1:200, ab183612) antibody was purchased from Invitrogen or Abcam. EEA1 (1:200, ab2900) antibody and Rab7 (1:200, ab50533) antibody were purchased from Abcam. LAMP1 (1:100, sc-20011) antibody was purchased from Santa Cruz Biotechnology. Alexa Fluor 568 (1:500) and Alexa Fluor 647 (1:500) secondary antibody was purchased from Abcam.

### Western blot

Cells were lysed with sample buffer and boiled for 10 min. Proteins were separated by sodium dodecylsulfate polyacrylamide gel electrophoresis and were transferred to nitrocellulose membranes. The membranes were incubated with primary antibodies at 4 °C overnight and after washing, membranes were incubated with secondary antibodies for 1 h at 37 °C. After washing, membranes were incubated with ECL kit reagents and visualized using a chemiluminescence instrument (ImageQuant LAS 4000, GE Healthcare). APP (1:1000) antibody was purchased from Abcam. β-actin (1:1000) antibody was purchased from Proteintech. Horseradish peroxidase-conjugated secondary antibody (1:10,000) was purchased from Promega.

### Interaction assay of protein and nanoparticle

10 μg GO or BGO (contain 10 μg GO) were added to 0.6 mg/mL purified APP or BACE1 protein which were purchased from Sino Biological, incubated at 4 °C for 2 h and centrifuged at 14000 g for 30 min. The supernatant was saved and the pellet was washed 3 times with PBS containing 3% tween 20. The protein solution, the supernatant and the pellet were boiled for 10 min in the SDS loading buffer, followed by SDS-PAGE and western blot with appointed antibodies 32. For preparation of BSA modified GO (BGO), 20 mg BSA and 10 mg GO were mixed and ultrasonicated for 1 h. The suspension was then centrifugated at 14000 g for 2 h to remove excess BSA. The precipitation was collected and resuspended in water by ultrasonication for 15 min.

### Enzyme-Linked Immunosorbent Assay

The hippocampus was homogenized by guanidine HCl extraction buffer 33. The total Ab42 and Ab40 concentrations of cell culture medium or hippocampus homogenate were detected by ELISA kit (purchased from CUSABIO) according to the manufacturer's instructions.

### Animals

10 months old female C57BL/6 mice were purchased from the Model Animal Research Center of Nanjing University. All animals were housed in temperature, humidity, and light controlled rooms, with water provided ad libitum. Animal welfare and experimental procedures were carried out in accordance with the Ethical Regulations on the Care and Use of Laboratory Animals of Anhui Medical University and were approved by the school committee for animal experiments.

### Mice administration

Mice were anesthetized with 3.0% sevoflurane carried by 100% oxygen in the small animal anesthesia machine (R500, RWD Life Science). Mice body temperature was maintained at 37 °C. Bilateral intracerebral injection was performed stereotactically at coordinates: 2.18 mm posterior, 2.30mm lateral, 2.10 mm ventral, referenced to the bregma (Desktop Digital Stereotaxic Instruments, RWD Life Science) 34. 2 μL of GO (10mg/mL) or BGO (with GO concentration at 10 mg/mL) was injected using a 5 μL glass syringe with a fixed needle (Neuros Syringes, Hamilton, Switzerland). The sham mice were subjected to bilateral intracerebral injection with normal saline. For CQ administration, mice were intraperitoneally injected with 60 mg/kg CQ.

### Surgical model

After GO, BGO or CQ administration, the mice were subjected to an intramedullary fixation surgery for tibial fracture under sevoflurane anesthesia as described in a previous study 27. In brief, mice were anesthetized with 3.0% sevoflurane carried by 100% oxygen. A skin incision was made below the knee, the tibia was exposed, and a 0.3 mm pin was inserted into its medullary cavity, achieving intramedullary fixation. Next, the bone was fractured at the midpoint using a surgical scissor. Lastly, the wound was sutured after necessary debridement. For pain management, a 2% lidocaine solution was applied locally before the incision, and 1% tetracaine hydrochloride mucilage was applied to the wound twice daily 27.

### Fear conditioning test

The test consists of a training phase prior to surgery and a test phase 1 day after surgery. One day prior to surgery, the mice were trained for fear conditioning to establish long-term memory. Each mouse was placed into the conditioning chamber for a 120 s accommodation period, followed by six pairs of conditional-unconditional stimuli and another 60 s of remaining in the conditioning chamber. One pair of conditional-unconditional stimuli consists of a 20 s, 70 dB sine wave tone (conditional stimulus), then a contextual interval of 25 s (trace interval), and finally a 2 s, 0.70 mA electrical footshock (unconditional stimulus). The pairs of conditional-unconditional stimuli were separated by random intervals 60 s. The context FCT reflects hippocampal-dependent memory. In the context test, the mice were simply placed back into the conditioning chamber for 5 min without a tone or footshock 27. The percentage freezing time (defined as the time in which mice had no movements except for respiration) was recorded by the Panlab Startle and Fear combined system with Packwin 2.0 software.

### Statistical analysis

Distribution normality was assessed using the Kolmogorov-Smirnov test. All data were analyzed by one-way measures analysis of variance with Turkey's post hoc test, two-tailed t test or Kruskal-Wallis test. Statistical analysis was performed using GraphPad Prism 7.0 software.

## Results and Discussion

### Synthesis and characterization of GO

**Figure 1:**
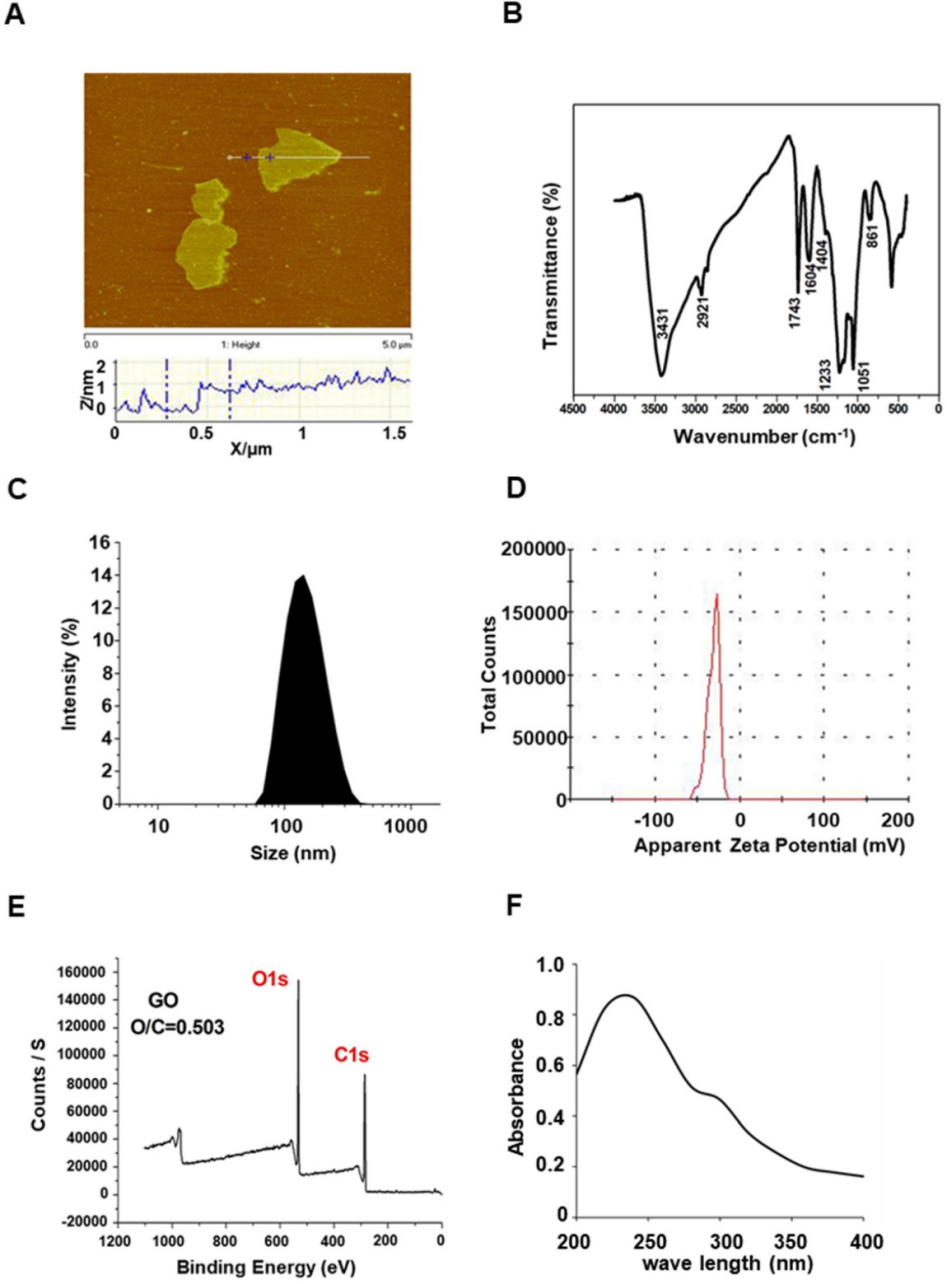
Characterization of GO. (A) AFM of GO. (B) FTIR spectra of GO. (C) Dynamic light scattering analysis of GO. (D) Zeta potential distribution of GO. (E) XPS spectra of GO. (F) UV-Vis spectrum of GO.

GO was prepared from natural graphite using the modified Hummers method 31. Atomic force microscopy (AFM) results showed that the thickness of the GO nanosheets was about 0.8-1.0 nm, indicating the formation of a single layer of GO (Figure 1A). The surface states of GO were characterized by Fourier-transform infrared (FTIR) spectroscopy. As shown in Figure 1B, the absorption band near 1743 cm^-1^ was attributed to the carboxyl group, while the absorption bands near 3431 cm^-1^ and 1604 cm^-1^ were attributed to O-H stretching and deformation vibration, indicating that the GO surface contains various chemical functional groups. In agreement with the AFM data, dynamic light scattering (DLS) assays showed an effective hydrodynamic size of approximately 200 nm in deionized water (Figure 1C), and exhibited a negative charge of -30.0 ± 2.06 mV (Figure 1D), leading to stable dispersions in water owing to electrostatic repulsion between particles. Different ratios of carbon to oxygen give different properties to those of GO. We used high-resolution X-ray photoelectron spectroscopy (XPS) to detect the surface concentrations of C and O. As shown in Figure 1E, the C 1s peak was found at ~287.56 eV, and the O 1s peak at ~535.19 eV, the oxygen to carbon ratio was 0.503. Consistent with previous reports 31, ultraviolet-visible (UV-VIS) spectroscopy revealed a broad optical absorption peak at 230 nm (Figure 1F).

### GO reduces intracellular Aβ

**Figure 2:**
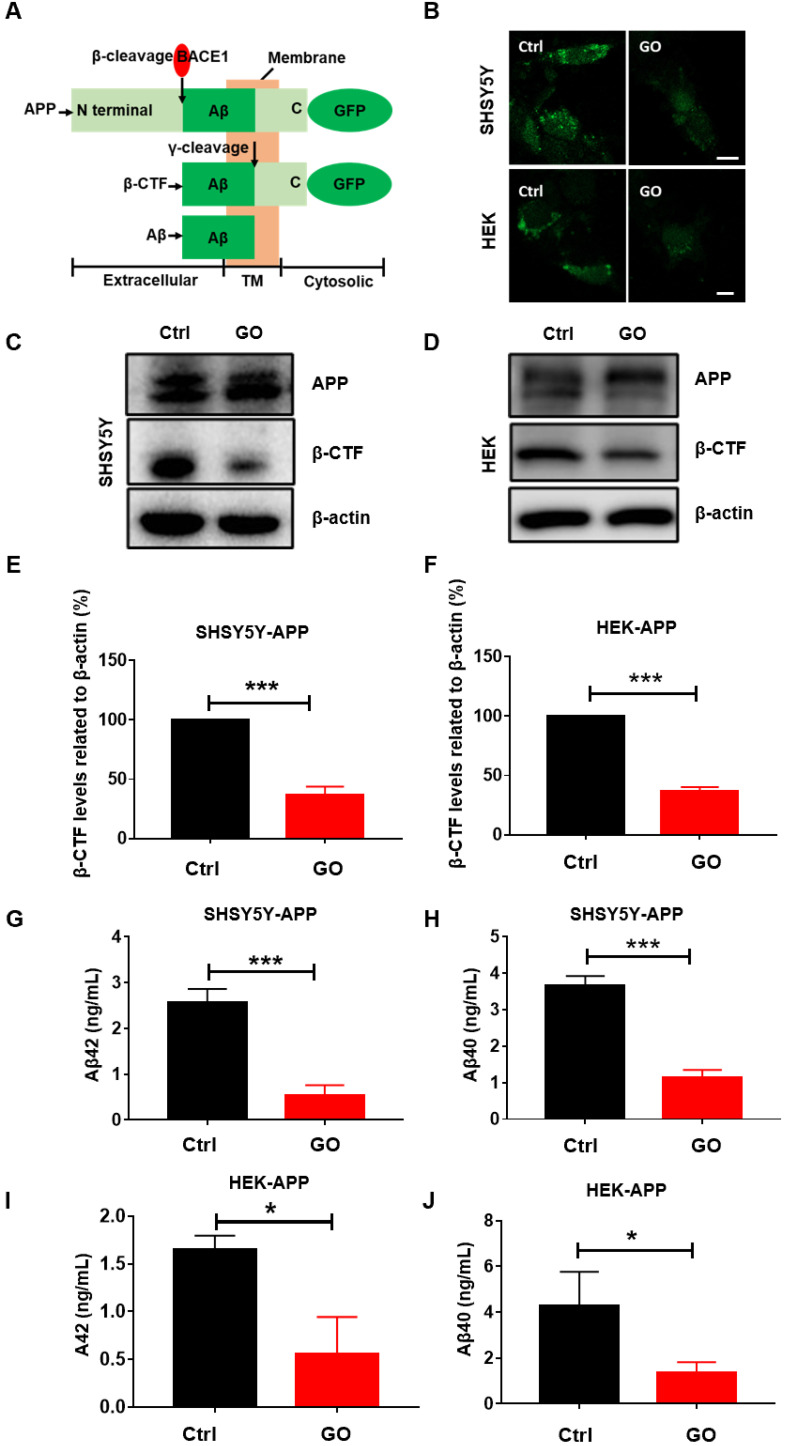
GO reduces intracellular level of Aβ. (A) Schematic representation of APP proteolytic processing indicating N-terminal (N) extracellular domain, transmembrane domain (TM), and C-terminal (C) cytosolic domain, with position of β cleavage sites, resulting β-CTF peptides. (B) Fluorescent image of SHSY5-APP-GFP (upper) and HEK293T-APP-GFP (bottom). Scale bar = 20 µm. (C) SHSY5-APP and (D) HEK293T-APP western blot results of APP, β-CTF and β-actin. (E) and (F) Statistical results of β-CTF in SHSY5-APP and HEK293T-APP cells. (G-J) ELISA results of aβ42 and aβ40 in SHSY5-APP and HEK293T-APP cells. Cells were treated with PBS or 60 µg/mL of GO for 24 h. Data are presented as the mean ± SEM, *p < 0.05, ** *p <* 0.01, *** *p <* 0.001.

To evaluate the effects of GO on Aβ levels, we established HEK293T-APP-GFP and SHSY5Y-APP-GFP cells, which are commonly used for the mechanistic study of Aβ 35, 36. In these cells, APP labeled with GFP at the C-terminus is overexpressed. As shown in Figure 2A, β-CTF and Aβ are generated by sequential β- and γ-cleavage of APP. APP and β-CTF with GFP at the C terminus were observed under fluorescence microscopy. We observed that the fluorescence signal was decreased in GO-treated cells, indicating a reduction of APP or β-CTF (Figure 2B). Western blot results showed that APP levels were not significantly different, but β-CTF levels were reduced in GO-treated cells (Figure 2C-F). After cleavage, β-CTF generates Aβ peptides, most of which are 40 (Aβ40) or 42 (Aβ42) residues in length. Aβ42 is more hydrophobic and more prone to fibril formation than Aβ40, and it is also the predominant isoform found in cerebral plaques 37. Both soluble and insoluble forms of Aβ have been found in cells 33, 38, and we detected the total content of Aβ42 and Aβ40. The results showed that GO significantly reduced the levels of Aβ42 and Aβ40 in both SHSY5Y and HEK293T cells (Figure 2G-J). Collectively, GO significantly alleviated the intracellular Aβ burden.

### Inhibited β-cleavage contributes to GO-decreased amyloid beta

**Figure 3:**
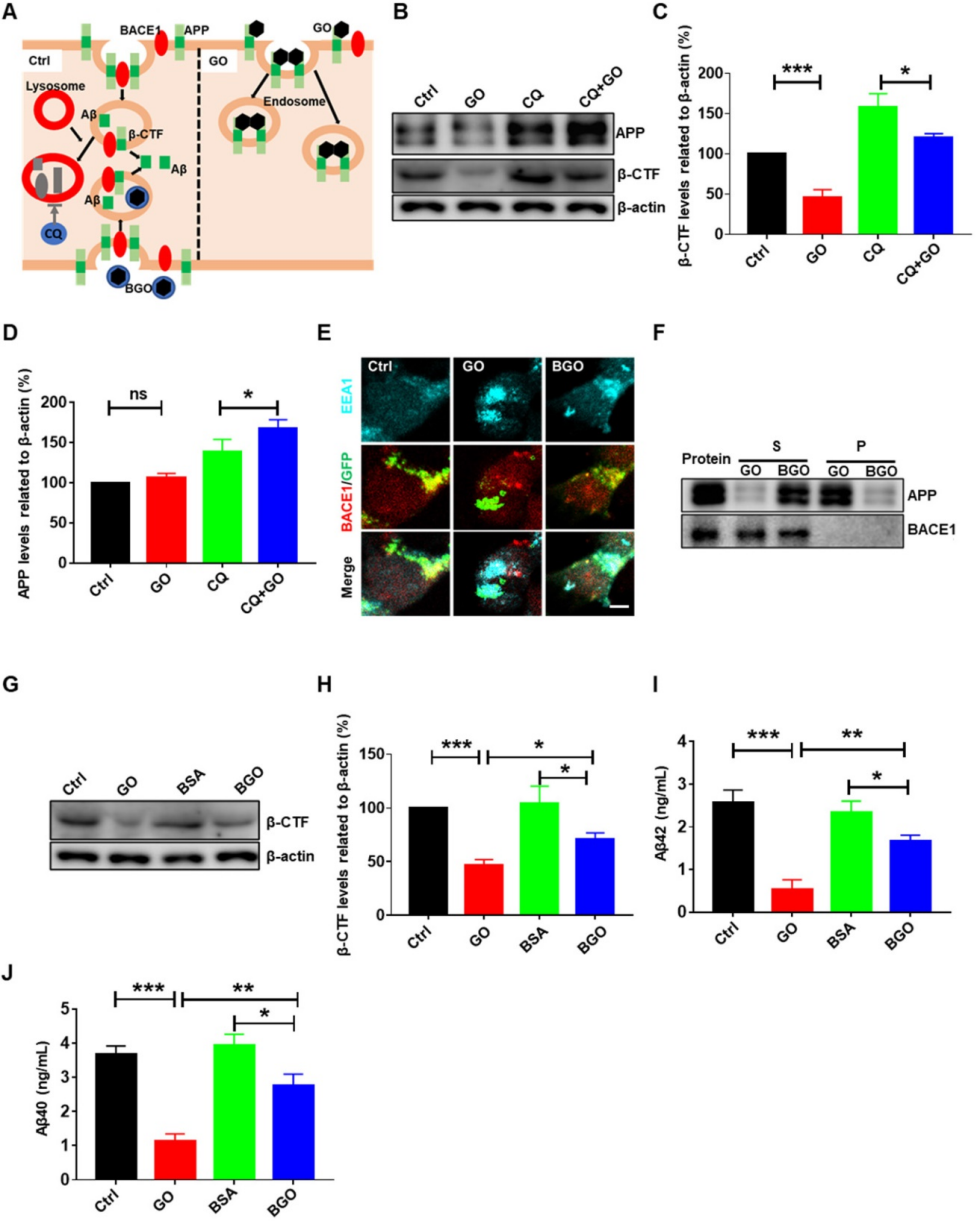
Inhibited β-cleavage contributes to GO-decreased Aβ. (A) Schematic diagram of β-cleavage contributing to Aβ production. APP is internalized into endosomes and cleaved by BACE1 to generates β-CTF. Then β-CTF is subsequently cleaved by γ-secretase generating Aβ peptides. Endosomes fuse with lysosomes to degrade their cargos, such as APP, β-CTF, and Aβ. CQ impairs the lysosomal degradation of APP, β-CTF, and Aβ by disrupting lysosomal function. GO induces the formation of many endosomes which are rich in APP but have less BACE1, resulting in the inhibition of β-cleavage. (B) Western blot results and (C, D) statistical results of β-CTF, APP and β-actin. (E) Immunofluorescent image of APP/ β-CTF (green), BACE1 (red) and EEA1 (early endosome, blue). Scale bar = 10 µm. (F) Interaction assay of GO/BGO and APP. Western blot results of APP and BACE1. Protein indicates APP or BACE1 protein solution. S and P are the supernatant and the pellet, respectively, derived from the precipitation. (G) Western blot results and (H) statistical results of β-CTF and β-actin. (I and J) ELISA results of aβ42 and aβ40. SHSY5-APP were treated with PBS, 60 µg/mL GO, BSA, BGO, CQ (50 µM) or their combination for 24 h. Data are presented as the mean ± SEM, *p < 0.05, ** *p <* 0.01, *** *p <* 0.001.

The decrease in intracellular Aβ levels induced by GO may be due to either the decline of Aβ production or the acceleration of Aβ degradation. First, we tested the production of Aβ. It is known that Aβ is generated from the endoproteolysis of APP though the amyloidogenic pathway in which APP is internalized into early endosomes and cleaved by BACE1 to generate β-CTF. Then, β-CTF is cleaved by γ-secretase to generate Aβ peptides (Figure 3A). Therefore, β-cleavage of APP is the rate-limiting step in the generation of Aβ peptides. We previously observed that β-CTF was reduced in GO-treated cells (Figure 2C-F). Accounting for that β-CTF maybe degraded by lysosome (Figure 3A), we use CQ to disrupt the degradation function of lysosome. Under CQ treatment, GO still decreased β-CTF, but increased APP levels (Figure 3B-D). These results suggested that the β-cleavage of APP was inhibited in the GO-treated cells.

BACE1 mediates the β-cleavage of APP; however, there were no significant changes in BACE1 expression levels after GO treatment (Figure S1). Thus, we further investigated the location of BACE1 in SHSY5Y-APP-GFP cells. EEA1 is a marker of early endosomes. We found that BACE1 co-localized with APP/β-CTF and early endosomes in control cells (Figure 3E). Interestingly, the co-localization of BACE1 and APP/β-CTF was reduced in GO-treated cells (Figure 3E), confirming the inhibited β-cleavage of APP by BACE1. Artificial materials are generally reported to induce endosome formation in which they are engulfed. Mass endosomes were consistently observed in GO-treated cells (Figure 3E). Additionally, as GO was shown to interact with Aβ 26, 39, we speculate that GO may also interact with its precursor protein APP, resulting in most endosomes co-localizing with APP, but rarely with BACE1 in GO-treated cells. Indeed, in the interaction assay of proteins and nanoparticles, most APP was detected in GO pellets, whereas BACE1 was only detected in the supernatant but not in GO pellets, suggesting that GO interacted with APP (Figure 3F). Bovine serum albumin (BSA) could block the affinity of GO; therefore, we constructed a BSA-modified GO (BGO) 40. Western blot results showed that APP was barely detectable in the BGO pellet; in contrast, it was enriched in the supernatant (Figure 3F). These results confirmed that BSA modification blocked the interaction between GO and APP. Moreover, we observed that BACE1 and APP/β-CTF co-localized in BGO-treated cells, similarly to the control cells (Figure 3E), demonstrating that the disrupted co-localization of APP and BACE1 was mediated by the interaction between GO and APP. Consequently, the disrupted co-localization of APP and BACE1 blocked the β-cleavage of APP, resulting in reduced β-CTF and decreased Aβ generation in GO-treated cells (Figure 3G-J). However, BSA modification restored GO-disrupted co-localization of APP and BACE1 and significantly increased GO-reduced Aβ levels (Figure 3E and G-J), demonstrating that inhibited β-cleavage contributed to GO-decreased Aβ generation.

### GO enhances lysosomal degradation of Aβ

**Figure 4:**
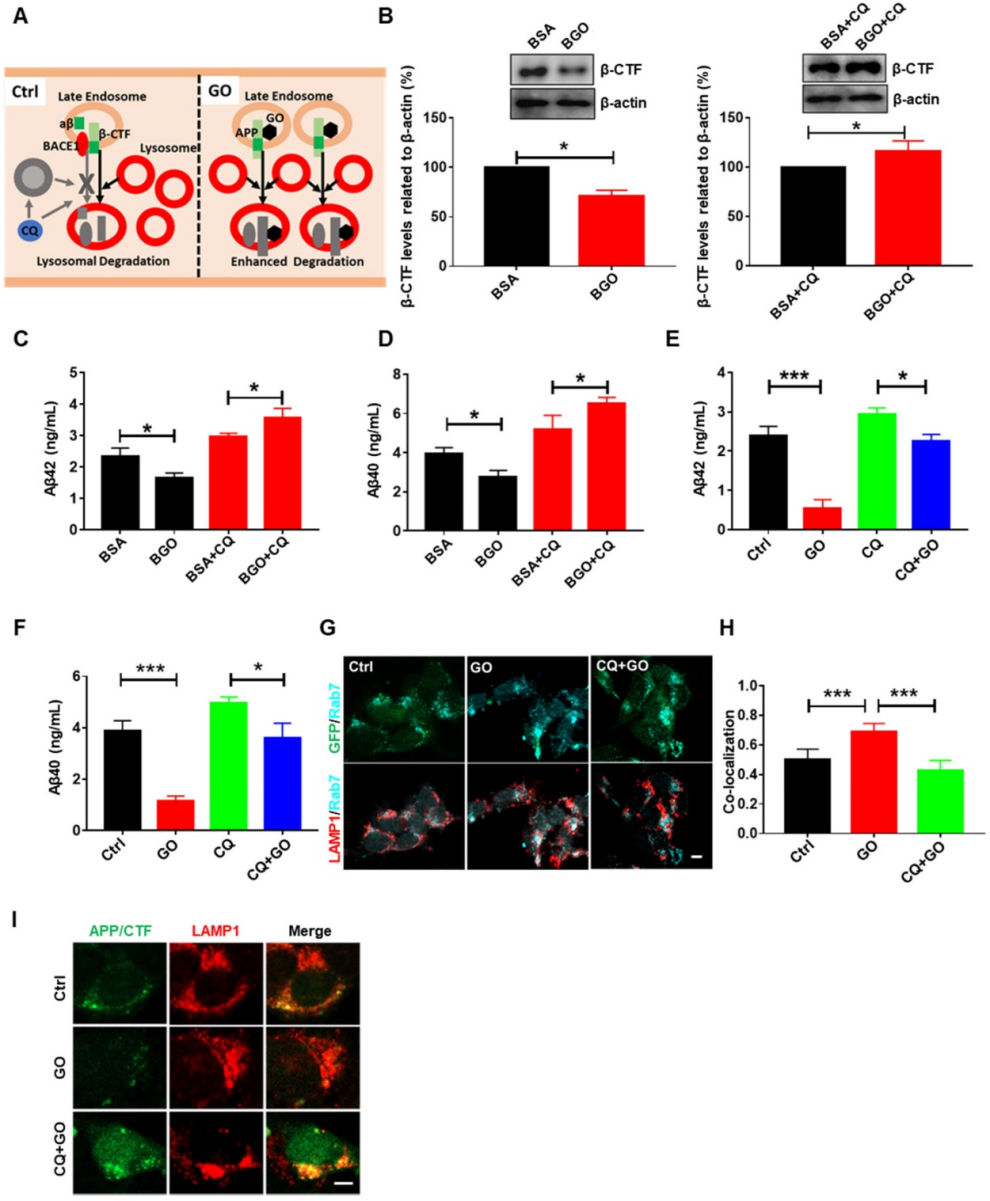
GO enhances the lysosomal degradation of Aβ. (A) Schematic diagram of enhanced lysosomal degradation of Aβ. Endosomes fuse with lysosomes to degrade their cargos, such as APP, β-CTF, and Aβ. CQ treatment impairs lysosomal degradation of APP, β-CTF, and Aβ by disrupting lysosomal function. GO induces the formation of many endosomes, improves the fusion of endosomes and lysosomes, and enhances the lysosomal degradation of APP, β-CTF and Aβ. (B) Western blot and statistical results of β-CTF and β-actin. (C-F) ELISA results of aβ42 and aβ40. (G) Immunofluorescent image of APP/β-CTF (green), LAMP1 (red) and Rab7 (blue). Scale bar = 10 µm. (H) Co-localization between Rab7 and LAMP1. (I) Immunofluorescent image of APP/β-CTF (green) and LAMP1 (red). Scale bar = 10 µm. SHSY5-APP were treated with PBS, 60 µg/mL GO, BSA, BGO, CQ (50 µM) or their combination for 24 h. Data are presented as the mean ± SEM, *p < 0.05, ** *p <* 0.01, *** *p <* 0.001, ns = no significant difference.

Although BSA modification significantly inhibited the reduction of β-CTF and Aβ levels by GO, the β-CTF and Aβ levels in BGO-treated cells were still significantly lower than those in BSA-treated cells (Figure 3G-J). These results indicate that other mechanisms may be involved in the decrease of Aβ by GO. As mentioned above, in addition to the decline in Aβ generation, the acceleration of Aβ degradation could also decrease the intracellular Aβ concentration, so we decided to test it. Early endosomes mature to late endosomes and fuse with lysosomes to degrade engulfed cargos, such as APP, β-CTF, and Aβ 41, 42 (Figure 4A). BGO was used to exclude the intervention of β-cleavage, and CQ was used to disrupt the degradation of lysosomes. We then compared the effects of BGO on β-CTF, Aβ42, and Aβ40 levels with or without CQ treatment. We found that the decrease in β-CTF, Aβ42, and Aβ40 by BGO were abrogated under the CQ treatment (Figure 4B-D), suggesting a contribution of lysosomal degradation to the decrease of Aβ by GO. Additionally, we also detected that GO significantly reduced β-CTF, Aβ42, and Aβ40 levels, but this reduction of Aβ was alleviated by CQ treatment (Figure 3B-C and 4E-F), indicating that lysosomal Aβ degradation was enhanced by GO. Notably, the inhibition of β-cleavage may also contribute to GO-reduced Aβ42 and Aβ40 in CQ-treated cells.

Next, to further confirm that GO can enhance Aβ degradation, we observed the abundance of APP/β-CTF in late endosomes and lysosomes. Rab7 is a marker protein of late endosomes. As shown in Figure 4G, a large number of APP/β-CTF were located in Rab7-labeled late endosomes. GO significantly increased the co-localization of Rab7 and LAMP1 (Figure 4G-H), indicating that GO treatment improved the delivery of endosomal cargo to lysosomes. In addition, less APP/β-CTF was observed in lysosomes in GO-treated cells than in control cells (Figure 4I), suggesting that Aβ degradation was enhanced by GO. Moreover, additional CQ treatment decreased Aβ delivery and lysosomal degradation (Figure 4G-I). Collectively, these results demonstrated that GO-enhanced lysosomal degradation was involved in GO-decreased Aβ levels. Notably, the present study did not detect Aβ degradation pathways beyond lysosomal degradation, such as proteolysis and glial cell-mediated clearance 43, which need to be further investigated.

### GO reduces hippocampal levels of Aβ in postoperative mice

**Figure 5:**
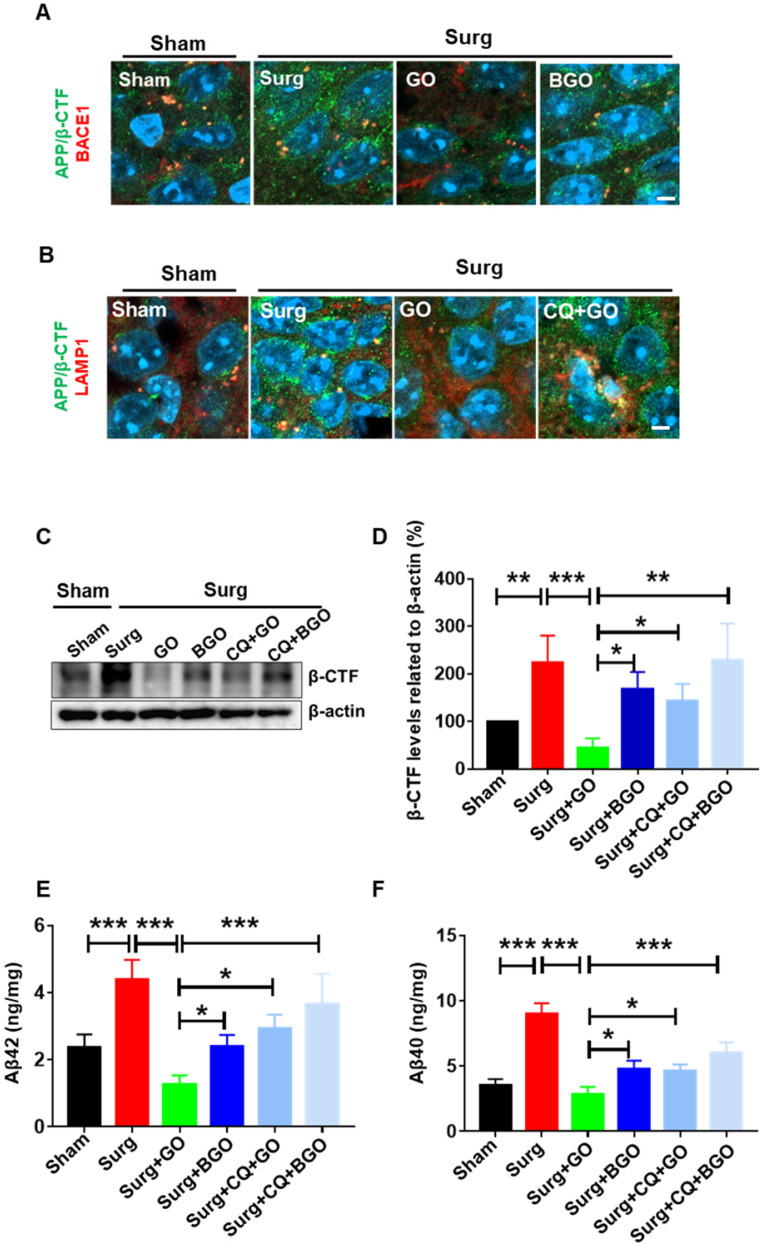
GO reduces hippocampal Aβ level in postoperative mice. (A-B) Immunofluorescent image of APP/β-CTF (green), BACE1 (red) and LAMP1 (red). Nucleus (blue) were stained with DAPI. Scale bar = 10 µm. (C) Western blot results and (D) statistical results of β-CTF and β-actin in postoperative mice hippocampus. (E-F) ELISA results of hippocampal aβ42 and aβ40. Data are presented as the mean ± SEM, *p < 0.05, ** *p <* 0.01, *** *p <* 0.001.

According to our results, GO can maximally decrease Aβ accumulation by simultaneously reducing Aβ generation and enhancing Aβ degradation *in vitro*; therefore, we hypothesized that GO would be beneficial for the treatment or prevention of Aβ-related diseases. Many clinical and basic studies have shown that the effects of Alzheimer's disease treatment through merely targeting Aβ clearance are limited 44. Therefore, a mouse model of POCD, which is a common central nervous system complication often accompanied by an acute increase in Aβ levels, was used in our study. Intramedullary fixation surgery for tibial fractures under inhalation anesthesia is commonly used to impair fear memory in rodents 27-29. In this model, we observed that hippocampal APP/β-CTF levels were obviously increased compared with those in sham mice (Figure 5A-B). Additionally, western blot and ELISA results showed that surgery and anesthesia significantly elevated β-CTF, Aβ42, and Aβ40 levels in the hippocampus (Figure 5C-F). These results demonstrated the induction of acute Aβ burden in postoperative mice. Before using GO as a therapeutic agent, we first tested the biocompatibility of GO. As shown in Figure S2-S4, the dosage of GO used in this study is relatively safe. Furthermore, we observed that GO administration resulted in less co-localization of APP/β-CTF and BACE1 and decreased lysosomal APP/β-CTF in the hippocampus of surgically treated mice; however, these effects were abrogated by BSA modification and additional CQ treatment, respectively (Figure 5A-B). These results indicated that GO inhibited β-cleavage and enhanced lysosomal degradation of APP *in vivo*. Consequently, GO administration inhibited the increase of β-CTF, Aβ42, and Aβ40 levels in the hippocampus of surgically treated mice, whereas BSA modification and additional CQ treatment significantly reversed the effects of GO (Figure 5C-F). Notably, a single administration of GO could not reduce the Aβ plaques (Figure S5).

### GO improves postoperative cognitive dysfunction in mice

**Figure 6:**
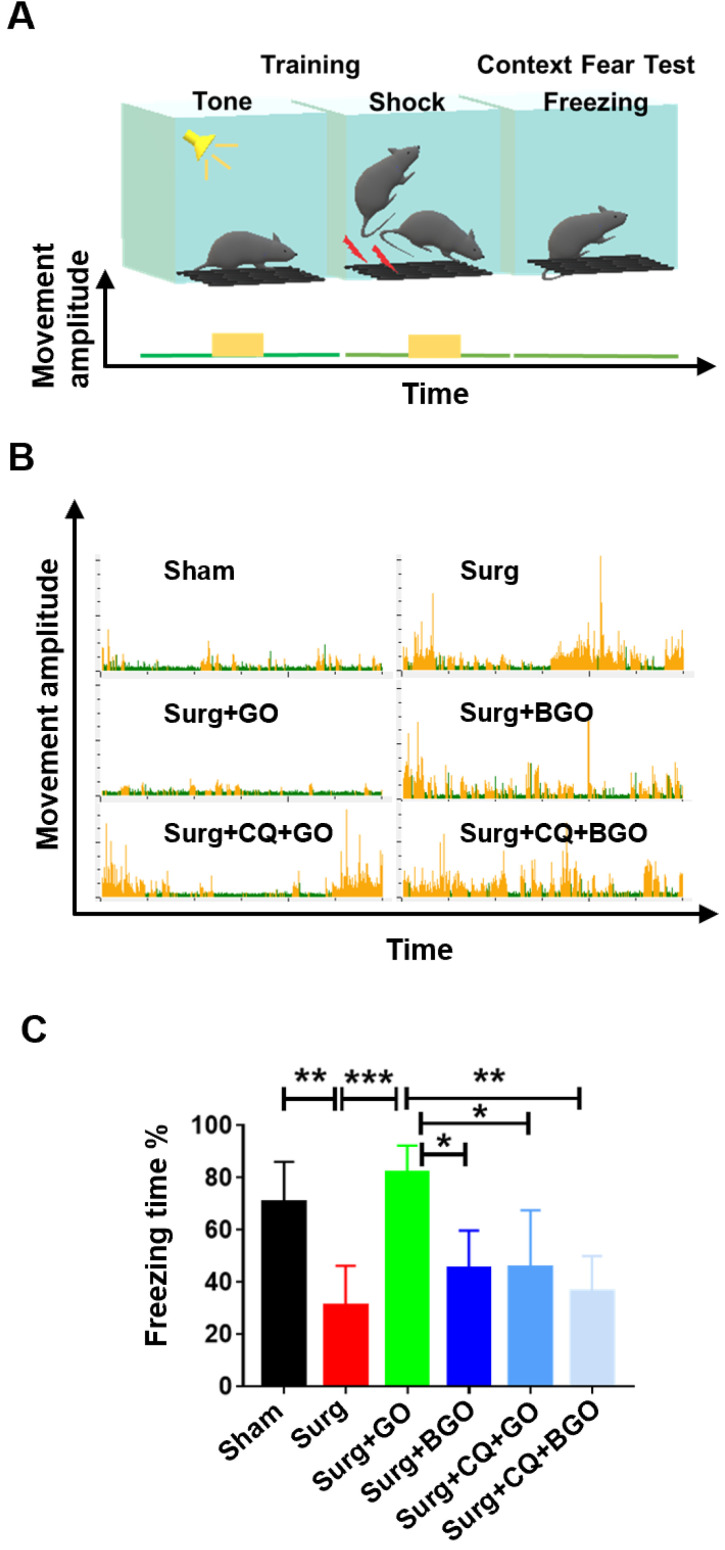
GO improves postoperative cognitive dysfunction in mice. (A) Schematic diagram of fear conditioning test. Tone or electrical footshock will induce high movement amplitude of mice. After training with the above stimulus, the mice will establish a fear memory. When the fear memory is evoked, they will exhibit freezing behavior, and very few changes in movement amplitude will be recorded. (B) Representative images of changes in mice movement amplitude. (C) The percentage of freezing time in the context test. Data are presented as the mean ± SEM, *p < 0.05, ** *p <* 0.01, *** *p <* 0.001.

Next, we assessed whether alleviation of the Aβ burden by GO could affect the behavior of post-surgery mice. Fear conditioning test (FCT) is commonly used to test the cognitive function of mice following fracture surgery 27-29. FCT consists of a training phase prior to surgery and a test phase 1 day after surgery. During the training phase, mice were stimulated with 70-dB sine wave tone and 0.70-mA electrical footshock to establish long-term memory. Then, a context test, which reflects hippocampal-dependent memory, was carried out in the test phase of the FCT. During training and testing, changes in gravity of the mice on the deck were recorded as movement amplitude (Figure 6A). When the fear memory of the mice was evoked, they exhibited freezing behavior, and very few changes in gravity were recorded. If the cognitive function of mice was impaired, no fear memory would be evoked and mice would be active, and continuous higher movement amplitudes could be detected (Figure 6A). As shown in Figure 6B, surgery-treated mice exhibited more activity than sham mice, indicating impaired fear memory. GO-treated mice showed prolonged freezing behavior compared to that of surgery-treated mice, and BGO-, additional CQ-, or combination-treated mice, which were more active than GO-treated mice (Figure 6B). Furthermore, the statistical results showed that surgery and anesthesia decreased the freezing time in the context test, while GO administration restored freezing time. BSA modification and additional CQ treatment significantly decreased the freezing time of GO-treated mice (Figure 6C). GO significantly decreased hippocampal Aβ, while BSA modification or additional CQ treatment restored Aβ. Therefore, these results demonstrated that GO improved the postoperative cognitive dysfunction of mice by alleviating the Aβ burden.

## Conclusion

In summary, GO treatment was found to maximally alleviate Aβ levels by simultaneously reducing Aβ generation and enhancing its degradation. Surgery and anesthesia acutely increased the hippocampal Aβ levels and impaired hippocampal-dependent fear memory in 10-month-old mice. However, GO administration significantly reduced hippocampal Aβ levels and improved the cognitive function of the mice. These results revealed that GO could improve POCD by maximally alleviating Aβ accumulation in mice, providing new evidence for the application of GO-based nanomedicines in Aβ-related diseases.

## Supplementary Material

Supplementary figures.
