# Supplementary material for: Graphene oxide improves postoperative cognitive dysfunction by maximally alleviating amyloid beta burden in mice
Source: Theranostics. 2020 Oct 25;10(26):11908–20. doi: 10.7150/thno.50616 (PMC7667672; doi:10.7150/thno.50616)
Supplement: Supplementary file 1 — Supplementary figures. [file thnov10p11908s1.pdf]

Supplementary Figure 1

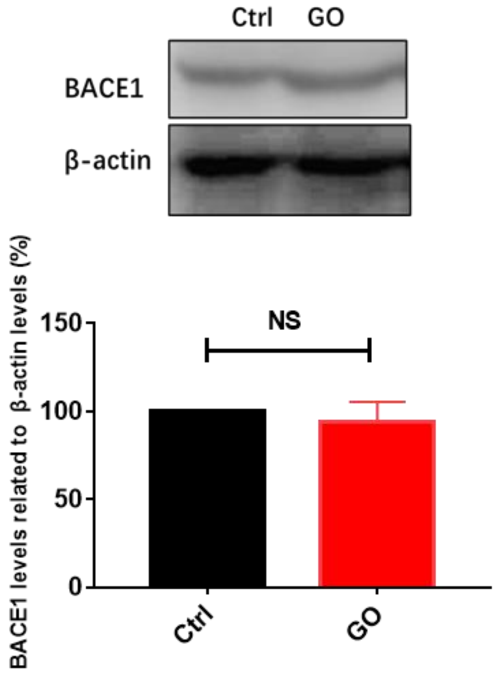

Fig S1 Western blot results and statistical results of BACE1 and β-actin. SHSY5-APP were treated with PBS or 60 μg/mL GO for 24 h. Data are presented as the mean ± SEM, NS = no significant difference.

Supplementary Figure 2

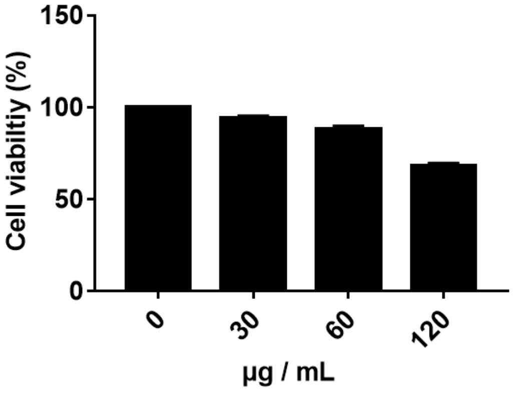

Fig S2 Cell viability of SHSY5-APP cells. Cell viability was determined using the CCK8 assay. SHSY5-APP were treated with 0, 30, 60 and 120 μg/mL GO for 24 h.

Supplementary Figure 3

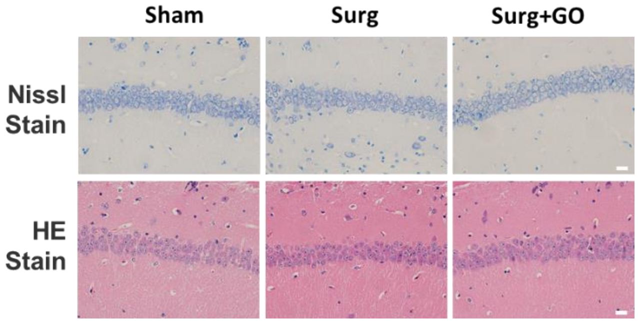

Fig S3 Nissl and HE staining of hippocampus. Sham or surgery mice pre-treated with normal saline solution or 2 μL GO. 24 hours after surgery, mice were sacrificed and brain were taken. Scale bar = 20 μm.

Supplementary Figure 4

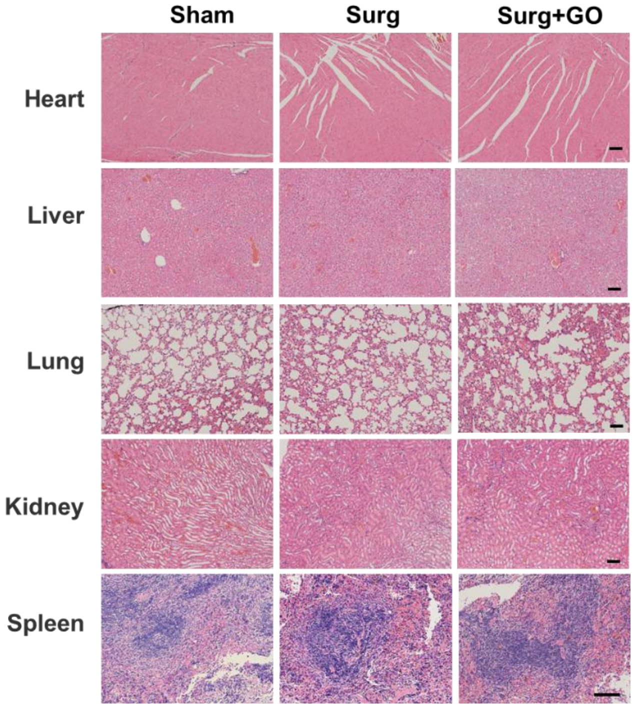

Fig S4 HE staining of peripheral organs. Sham or surgery mice pre-treated with normal saline solution or 2 μL GO intracerebrally. 24 hours after surgery, mice were sacrificed and the main peripheral organs were taken. Scale bar = 100 μm.

Supplementary Figure 5

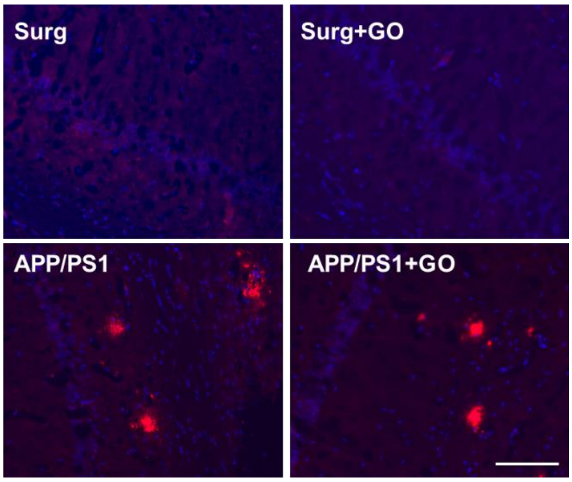

Fig S5 Immunofluorescence of Aβ plaque. 10 months old surgery mice or 7 months old APP/PS1 mice were treated with normal saline solution or 2 μL GO. 24 hours after surgery, mice were sacrificed and brain were taken. Brain sections were immunostained with the anti-Aβ antibody and the nuclei were stained with Hoechst. Scale bars = 100 μm.
